# H2Opred: a robust and efficient hybrid deep learning model for predicting 2’-O-methylation sites in human RNA

**DOI:** 10.1093/bib/bbad476

**Published:** 2024-01-04

**Authors:** Nhat Truong Pham, Rajan Rakkiyapan, Jongsun Park, Adeel Malik, Balachandran Manavalan

**Affiliations:** Department of Integrative Biotechnology, College of Biotechnology and Bioengineering, Sungkyunkwan University, Suwon, 16419, Republic of Korea; Department of Mathematics, Bharathiar University, Coimbatore - 641046, Tamil Nadu, India; InfoBoss inc. and InfoBoss Research Center, Gangnam-gu, Seoul 06278, Republic of Korea; Institute of Intelligence Informatics Technology, Sangmyung University, Seoul, 03016, Republic of Korea; Department of Integrative Biotechnology, College of Biotechnology and Bioengineering, Sungkyunkwan University, Suwon, 16419, Republic of Korea

**Keywords:** 2’-O-methylation sites, convolutional neural network, gated recurrent unit, hybrid deep learning, bioinformatics, natural language processing

## Abstract

2’-O-methylation (2OM) is the most common post-transcriptional modification of RNA. It plays a crucial role in RNA splicing, RNA stability and innate immunity. Despite advances in high-throughput detection, the chemical stability of 2OM makes it difficult to detect and map in messenger RNA. Therefore, bioinformatics tools have been developed using machine learning (ML) algorithms to identify 2OM sites. These tools have made significant progress, but their performances remain unsatisfactory and need further improvement. In this study, we introduced H2Opred, a novel hybrid deep learning (HDL) model for accurately identifying 2OM sites in human RNA. Notably, this is the first application of HDL in developing four nucleotide-specific models [adenine (A2OM), cytosine (C2OM), guanine (G2OM) and uracil (U2OM)] as well as a generic model (N2OM). H2Opred incorporated both stacked 1D convolutional neural network (1D-CNN) blocks and stacked attention-based bidirectional gated recurrent unit (Bi-GRU-Att) blocks. 1D-CNN blocks learned effective feature representations from 14 conventional descriptors, while Bi-GRU-Att blocks learned feature representations from five natural language processing-based embeddings extracted from RNA sequences. H2Opred integrated these feature representations to make the final prediction. Rigorous cross-validation analysis demonstrated that H2Opred consistently outperforms conventional ML-based single-feature models on five different datasets. Moreover, the generic model of H2Opred demonstrated a remarkable performance on both training and testing datasets, significantly outperforming the existing predictor and other four nucleotide-specific H2Opred models. To enhance accessibility and usability, we have deployed a user-friendly web server for H2Opred, accessible at https://balalab-skku.org/H2Opred/. This platform will serve as an invaluable tool for accurately predicting 2OM sites within human RNA, thereby facilitating broader applications in relevant research endeavors.

## INTRODUCTION

The epitranscriptome involves chemical modification to messenger RNA (mRNA), influencing gene expression [1]. While current epitranscriptome research has focused on base modifications, the ribose sugar can also be methylated at the 2′ position to form 2’-O-methylated nucleotides (2OM) [2]. 2OM has been found in all types of RNA, including mRNA, ribosomal RNA (rRNA), transfer RNA (tRNA), micro RNA (miRNA), small nucleolar RNA (snoRNA) and PIWI-interacting RNA (piRNA) [3]. The process of 2OM can be achieved through two distinct enzymatic routes: standalone methyltransferases and RNA-protein complex guided by Box C/D snoRNA, known as C/D-box snoRNPs. Standalone methyltransferases recognize their targets based on sequence and shape, while C/D-box snoRNPs are guided to their targets by snoRNAs [4–6]. Consequently, 2OM can occur in all four canonical nucleotides (adenine, cytosine, guanine and uracil) and some non-standard nucleotides.

Recent studies have shown that 2OM not only stabilized RNA but also involved in regulating gene expression and other cellular processes. For example, 2OM in RNA can be used by the innate immune system to distinguish between internal and external mRNA [6]. HIV-1 evades detection by the MDA5 protein using the TRBP-FTSJ3 complex to add 2OM to its RNA [7]. In human monocytes, 2OM RNA can suppress the release of pro-inflammatory cytokines [8]. In yeast, specific 2OM sites in telomerase RNA affect how well telomerase functions [9]. 2OM is also important for antibiotic resistance and cancer. However, much remains to be learned about the full range of roles that 2OM plays in RNA. Identifying 2OM sites in RNA is essential for understanding its functional significance, which is one of the important research topics in the community.

Several experimental methods have been developed to accurately identify 2OM in RNA. These methods include perchloric acid (HClO4) hydrolysis, periodate oxidation hydrolysis, chromatography and mass spectrometry. However, these methods are labor-intensive, need specialized tools, can damage RNA samples and are challenging to use with minimal RNA. To overcome these limitations, high-throughput techniques based on deep sequencing have also been developed, such as Nm-seq [10], RiboMeth-seq [11], 2OMe-seq [12], RibOxi-Seq [13] and Nm-seq (a) [14]. While these methods can identify 2OM at the transcriptome level, they are still costly, time-consuming and require specialized expertise. Therefore, computational methods have been developed to complement the experimental methods.

Sun *et al*. [15] developed RMBase, a database that contained 18 independent high-throughput sequencing data for studying RNA post-transcriptional modifications. The updated version, RMBase v2.0 [16], contains data on 5096 2OM positions across three species, a significant increase from its predecessor’s 1209 entries. These databases serve as the foundation for developing computational methods to identify 2OM sites. Chen *et al*. [17] constructed the first benchmarking dataset based on RMBase and developed a support vector machine (SVM)-based predictor. Using the same dataset, three more methods were proposed: iRNA-2OM [18], identification of RNA 2OM using pseudo k-tuple nucleotide composition (iRNA-PseKNC(2methyl)) [19] and an ensemble model by Huang *et al*. [20]. Later, Zhou’s group developed two tools, NmSEER [21] and NmSEER V2.0 [22], based on different approaches and datasets. Li *et al*. [23] proposed DeepOMe based on convolutional neural network (CNN) and bidirectional long short-term memory (Bi-LSTM) layers. Ao *et al.* [24] developed NmRF using Chen’s dataset and RMBase v2.0, which achieved an excellent performance on three species. Yang *et al*. [25] introduced i2OM by constructing a larger dataset based on *Homo sapiens.* Their study showed better performance using conventional machine learning (ML) approaches and two-step feature selection techniques. While these methods have made significant contributions to the field, there is still room for improvement.

In this study, we introduced H2Opred, a novel hybrid deep learning (HDL) model, for accurately identifying 2OM sites. Remarkably, this is the first application of HDL in developing nucleotide-specific models [adenine (A2OM), cytosine (C2OM), guanine (G2OM) and uracil (U2OM)] as well as a generic model that integrated all nucleotide-specific datasets (N2OM). H2Opred included all these five models, each of which has been trained in a similar fashion. H2Opred leveraged a set of 14 conventional descriptors, encompassing binary profile features (BPF), nucleotide chemical properties (NCP), dinucleotide binary encoding (DBE), as well as type 1 and type 2 dinucleotide physicochemical properties (DPCP_1, DPCP_2), positional specifics of two nucleotides (PS2), Kmer, reverse complement Kmer patterns (RCKmer), electron–ion interaction pseudo potentials of trinucleotides (PseEIIP), composition of k-spaced nucleic acid pairs (CKSNAP), enhanced nucleic acid composition (ENAC), Z curve parameters capturing frequencies of phase-specific trinucleotides (Zcurve), a novel combination of adaptive skip dinucleotide composition with local position-specific dinucleotide frequency (ASLPN) and multivariate mutual information combined with accumulated nucleotide frequency (MMNF). Additionally, five natural language processing (NLP)-based embeddings, including the DNA-language model (DNABERT), sequence-to-vector (Seq2Vec), word-to-vector (Word2Vec), FastText and global vectors for word representation (GloVe), were utilized. All of these descriptors and embeddings were directly extracted from RNA sequences. H2Opred incorporated both stacked 1D convolutional neural network (1D-CNN) blocks and stacked attention-based bidirectional gated recurrent unit (Bi-GRU-Att) blocks to learn effective feature representations from 14 conventional descriptors and five NLP-based embeddings, respectively. These feature representations were then combined to make the final prediction. Ablation analysis showed that both 1D-CNN and Bi-GRU-Att blocks were essential for H2Opred’s optimal performance. The H2Opred models exhibited outstanding performance during cross-validation and independent testing, surpassing the capabilities of large-scale conventional ML-based single-feature models. Notably, the generic H2Opred model outperformed both the existing predictor and the four nucleotide-specific H2Opred models, demonstrating that the intelligent integration of conventional descriptors and pretrained model-based embeddings extracted from a large training dataset through HDL enhances both prediction accuracy and interpretability.

## MATERIALS AND METHODS

### Dataset

We used the same benchmark dataset proposed by Yang *et al*. [25], because it is the most recent, comprehensive and non-redundant dataset available. The authors obtained 2OM sites from two different sources: RMBase v2.0 [16] and the recent experimental dataset generated by Nm-seq [10] and deposited in the GEO database (GSE90164). In total, they obtained 7597 2OM sites distributed in CDS, 3’-UTR, 5’-UTR, intron, exon and intergenic regions, and containing almost all types of RNA (tRNAs, rRNAs, scRNAs, scaRNAs, snRNAs, snoRNAs, lincRNAs, protein-coding genes, pseudogenes, etc.). Since the majority of sequences from RMBase v2.0 have a sequence length of 41 bp (20 upstream and 20 downstream based with the center 2OM sites), the authors extracted the same sequence length from the experimental data. To generate negative samples, the authors employed the same strategies as previous studies and generated a large number of sequences. Subsequently, they applied CD-HIT [26] with a threshold of 80% to remove redundant sequences, resulting in 6091 positive samples and 21 520 negative samples. To address class bias, which is a common problem when developing prediction models with imbalanced datasets [27], the authors selected the same number of negative samples as positive samples. The authors then divided the dataset into four subsets, centered on A, U, C and G corresponding to A2OM, U2OM, C2OM and G2OM, respectively. The A2OM dataset contains 2176 training and 934 testing samples, the U2OM dataset contains 2236 training and 960 testing samples, the C2OM dataset contains 2278 training and 788 testing samples and the G2OM dataset contains 1832 training and 788 testing samples. Notably, all training and testing samples contain an equal number of positive and negative samples. It should be noted that we have used the same training datasets for the model development in this study. However, we used entirely different testing datasets to check the model transferability. Specifically, we supplemented the existing nucleotide-specific testing datasets with our own collections. Positive samples were collected from RMDisease v2.0 [28], and excluded the redundant samples, resulting in 34, 82, 12 and 173 positive samples for A2OM, C2OM, G2OM and U2OM, respectively. Regarding the negative samples, we considered all possible chromosomes in *H. sapiens* and selected sequences with four different nucleotide bases as central residues, flanked by 20 nucleotides both upstream and downstream. From these, we randomly chose samples 10-fold larger in number than positive samples. Importantly, newly constructed samples were not present in the existing training and the testing datasets. Finally, we obtained 501 positive and 5010 negative samples for A2OM, 571 positive and 5710 negative samples for C2OM, 406 positive and 4060 negative samples for G2OM and 653 positive and 6530 negative samples for U2OM.

### Feature extraction

We used 14 conventional descriptors and five NLP-based embeddings to represent RNA sequences. The conventional descriptors covered different properties of sequence information and were widely used in bioinformatics [18, 29, 30]. The NLP-based embeddings were trained on large datasets of DNA and RNA sequences to capture their linguistic features. The conventional descriptors included Kmer, BPF, NCP, DBE, DPCP_1, DPCP_2, PS2, RCKmer, PseEIIP, CKSNAP, ENAC, Zcurve, ASLPN and MMNF. The NLP-based embeddings included DNABERT, Seq2Vec, Word2vec, FastText and GloVe. A detailed description of these descriptors and embeddings is provided in the supplementary information.

### Framework of H2Opred

**Figure 1:**
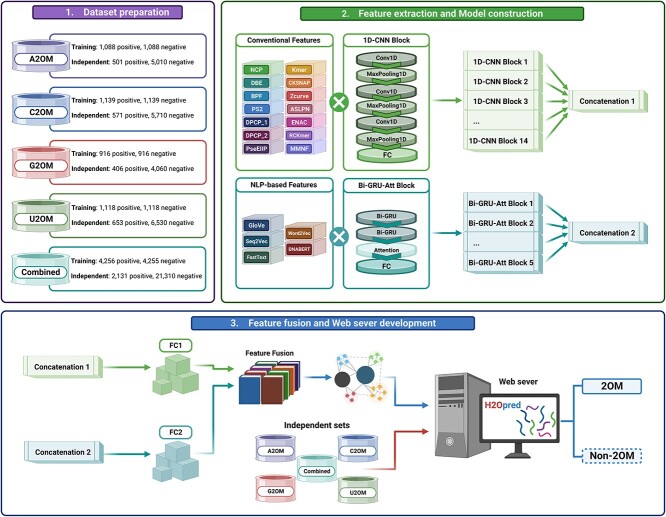
The workflow of constructing H2Opred framework. (A) Four 2’-O-methylation (2OM) site types were collected and split into training and independent datasets, where the combined dataset was constructed by integrating all four 2OM site types together as well as removing redundancy and identical samples. (B) Subsequently, 14 conventional descriptors and five NLP-based embeddings were extracted and then fed into stacked convolutional neural network (1D-CNN) blocks and stacked attention-based bidirectional gated recurrent unit (Bi-GRU-Att) blocks to acquire spatial and temporal-sequential feature representations for final prediction model. (C) During the testing phase, the testing datasets were submitted to the web server, resulting in either 2OM or non-2OM sites. (Created with BioRender.com).

In this study, we proposed an HDL model to predict 2OM sites in human RNA sequences (Figure 1). The HDL model consisted of stacked 1D-CNN blocks and stacked Bi-GRU-Att blocks, which learned and extracted both spatial and temporal-sequential feature representations from conventional descriptors and NLP-based embeddings. The details of these blocks are presented below.

### Stacked 1D-CNN

To extract and learn the spatial feature representations from the RNA sequences, we designed a 1D-CNN block for each conventional descriptor. Inspired by the stacking CNN approach utilized in recent studies [31–34], we developed 14 1D-CNN blocks and integrated them to handle the 14 conventional descriptors. Each 1D-CNN block consisted of three 1D convolutional (Conv1D) layers, three max pooling layers and one fully connected (FC) layer at the end. Given an input $X{c}_{in}$ of ($B,{L}_{in},{C}_{in}$), Conv1D will obtain an output $X{c}_{out}$ of ($B,{L}_{out},{C}_{out}$) as follows:


(1) \begin{equation*} X{c}_{ou t}\left(B,{C}_{{ou t}_j}\right)= bc+\sum \limits_{k=0}^{C_{in}-1} Wc\left({C}_{ou{t}_j},k\right)\ast X{c}_{in}\left(B,k\right), \end{equation*}

where $\ast$ denote the cross-correlation operation, $Wc$ and $bc$ are weight and bias, $B$ is the batch size, ${L}_{in}$, ${C}_{in}$, ${L}_{out}$ and ${C}_{out}$ are the input length, the number of the input channels, output length and the number of the output channels, respectively, in which ${L}_{out}$ can be computed as follows:


(2) \begin{equation*} {L}_{out}=1+\frac{L_{in}+2P-D\left(k-1\right)-1}{S}, \end{equation*}

where $P$, $D$, $\kappa$ and $S$ are the padding size, dilation rate, kernel size and stride, respectively. Then, each Conv1D layer was passed through a ReLU (rectified linear unit) activation function, which can be defined as follows:


(3) \begin{equation*} \mathrm{ReLU}(x)=\max \left(0,x\right). \end{equation*}

To achieve dimensional reduction, we incorporated one max pooling layer immediately after each Conv1D layer. The operation of each max pooling layer is outlined as follows:


(4) \begin{equation*} X{m}_{out}\left(B,{C}_j,k\right)=\underset{p=0,...,\kappa -1}{\max }X{m}_{in}\left(B,{C}_j,S\times k+p\right), \end{equation*}

where $X{m}_{in}$ and $X{m}_{out}$ are the input and output of the max pooling layer. It should be noted that only the length of the output changed after applying the max pooling layer. Finally, the feature representations were flattened and fed into an FC layer to produce the final feature representations. In addition, a dropout layer was applied after flattening the features to prevent overfitting. The configuration of each stacked 1D-CNN block was described in detail in the optimization process section.

### Stacked Bi-GRU-Att

To extract the temporal-sequential information from RNA sequences, we designed a Bi-GRU-Att block to capture temporal-sequential feature representations from each of five NLP-based embeddings extracted from RNA sequences. It should be noted that Bi-GRU was chosen over a single GRU because it can capture temporal-sequential feature representations in both the forward and backward directions of the sequence, resulting in more robust and informative feature representations. Additionally, Bi-GRU architecture was selected over LSTM or Bi-LSTM because it is simple and has fewer output gates, making it faster and more effective to train, especially with limited data. To address the challenge of not every feature representation equally informative for characterizing RNA sequence, we incorporated an attention mechanism [35–38] to selectively focus on the most relevant and crucial feature representations for discriminating 2OM sites. The mathematical equations of each GRU can be expressed as follows:


(5) \begin{equation*} {u}_t=\sigma \left({W}_{iu}{x}_t+{U}_{hu}{h}_{\left(t-1\right)}+{b}_{iu}+{b}_{hu}\right), \end{equation*}

(6) \begin{equation*} {r}_t=\sigma \left({W}_{ir}{x}_t+{U}_{hr}{h}_{\left(t-1\right)}+{b}_{ir}+{b}_{hr}\right), \end{equation*}

(7) \begin{equation*} \qquad\quad{\tilde{\eta}}_t=\tau \left({W}_{i\tilde{\eta}}{x}_t+{r}_t\odot \left({U}_{h\tilde{\eta}}{h}_{\left(t-1\right)}+{b}_{h\tilde{\eta}}\right)+{b}_{i\tilde{\eta}}\right), \end{equation*}

(8) \begin{equation*} {h}_t=\left(1-{u}_t\right)\odot{\tilde{\eta}}_t+{u}_t\odot{h}_{\left(t-1\right)},\qquad\quad \end{equation*}

where ${u}_t$, ${r}_t$, ${\tilde{\eta}}_t$ and ${h}_t$ represent the update gate, the reset gate, the new gate (or the hidden candidate status) and the hidden gate, respectively; ${x}_t$, $W$, $U$ and $b$ are the input vectors, the weight matrices and the bias vector; $\sigma$ denotes the sigmoid function, $\tau$ denotes the tanh function and $\odot$ denotes the Hadamard product.

And the mathematical equations of attention mechanism can be defined as follows:


(9) \begin{equation*} {A}_{ij}={x}_a^T\tau \left({W}_a{h}_i+b\right), \end{equation*}

(10) \begin{equation*} {\alpha}_{ij}=\frac{e^{\left({A}_{ij}\right)}}{\sum \limits_{t=1}^T{e}^{\left({A}_{ij}\right)}},\qquad\ \ \end{equation*}

(11) \begin{equation*} {c}_i=\sum \limits_{j=1}^T{\alpha}_{ij}{h}_j, \qquad\ \ \end{equation*}

where ${x}_a$ is the random initialization vector, ${A}_{ij}$ is the attention weight, ${h}_i=\left[{\overrightarrow{h}}_t;{\overleftarrow{h}}_t\right]$ is the output of Bi-GRU at the time step $t$ that concatenates both forward and backward information, $T=41$ is the length of the input sequence, ${\alpha}_{ij}$ is the output score of the attention weight by applying SoftMax function and ${c}_i$ is the attention output.

Motivated by the stacking recurrent neural network-based approaches employed in recent studies [39, 40], we developed five Bi-GRU-Att blocks that were subsequently combined to handle the five NLP-bassed embeddings. Notably, each Bi-GRU-Att block contained two Bi-GRU layers, followed by an attention layer and an FC layer. The hidden state generated by the first Bi-GRU layer was used as the input of the second Bi-GRU layer. For more details on the specific configuration of the Bi-GRU-Att blocks, please refer to the optimization process section.

### Optimization process of H2Opred

We conducted a comprehensive optimization process to select the best model for constructing H2Opred. Table S1 represents our parameter search range implemented in this study. Importantly, we used 5-fold cross-validation during the training phase to tune the hyperparameters within the specified search range and determine the optimal set of hyperparameters.

Here, the binary cross-entropy loss function (${L}_{BCE}$) was used as an objective function to optimize the parameters during training model, which can be defined as follows:


(12) \begin{equation*} {L}_{BCE}=\frac{1}{N_s}\sum \limits_{s=1}^{N_s}{y}_s\log \left(\wp \left({\hat{y}}_s\right)\right)+\left(1-{y}_s\right)\log \left(1-\wp \left({\hat{y}}_s\right)\right), \end{equation*}

where ${N}_s$ is the number of samples, $s=\mathrm{1...}{N}_s$, ${y}_s$ is the ground truth label and ${\hat{y}}_s$ is the predicted label. In addition, it should be noted that $\wp \left({\hat{y}}_s\right)$ represents the probability of 2OM and $1-\wp \left({\hat{y}}_s\right)$ represents the probability of non-2OM.

### Performance evaluation metrics

In this study, several commonly used performance evaluation metrics [41–43] were utilized to evaluate the model performance during training, independent testing as well as comparison, which included accuracy (ACC), Mathews correlation coefficient (MCC), sensitivity (Sn), specificity (Sp), precision (PRE), area under the receiver operating characteristic curve (AUC) and F1-score. Their mathematical equations can be expressed as follows:


(13) \begin{equation*} ACC=\frac{TP+ TN}{TP+ TN+ FP+ FN}, \end{equation*}

(14) \begin{equation*} MCC=\frac{TP\times TN- FP\times FN}{\sqrt{\left( TP+ FP\right)\times \left( TP+ FN\right)\times \left( TN+ FP\right)\times \left( TN+ FN\right)}}, \end{equation*}

(15) \begin{equation*} Sn=\frac{TP}{TP+ FN}, \end{equation*}

(16) \begin{equation*} Sp=\frac{TN}{TN+ FP}, \end{equation*}

(17) \begin{equation*} PRE=\frac{TP}{TP+ FP}, \end{equation*}

(18) \begin{equation*} F1- score=\frac{2\times TP}{2\times TP+ FP+ FN}, \end{equation*}

where TP represents the count of test results that correctly match with positive samples, TN represents the count of test results that correctly match with negative samples, FP stands for the count of test results that incorrectly indicate positive samples and FN denotes the count of test results that incorrectly indicate negative samples.

## RESULTS AND DISCUSSION

### Assessment of conventional descriptors using 11 different ML algorithms on the training and independent testing datasets

Inspired by previous studies [29, 44], we evaluated 14 conventional RNA sequence descriptors that capture different aspects of sequence information, such as composition, position-specific, physicochemical properties and sequence order. To quantify the intrinsic capability of these feature descriptors for distinguishing 2OM from non-2OM in different datasets, we evaluated the performance of each descriptor using 11 conventional ML-based classifiers (including random forest (RF), extremely randomized trees (ERT), gradient boosting trees (GBT), AdaBoost trees (ABT), extreme GBT (XGBT), SVM, deep neural network (DNN), light GBT (LGBT), decision trees (DT), logistic regression (LR) and catBoost (CB)) by means of randomized 10-fold cross-validation procedure. This technique helps to prevent model overfitting during training [45, 46].

**Figure 2:**
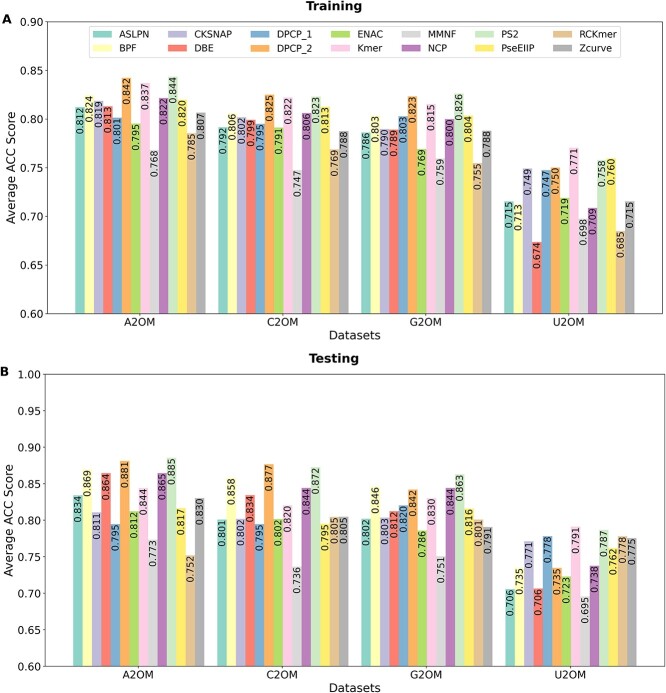
Average accuracy (ACC) achieved by 11 conventional ML-based classifiers for each of the 14 conventional descriptors. The comparative performance of four nucleotide-specific models is shown for training datasets (A) and testing datasets (B).

In total, we generated 616 models (154 models * 4 datasets) during the training and assessed its transferability with the independent testing datasets (Figures S1–S4). We were more interested in the discriminative capability of the feature descriptors than in the performance of any specific classifier. To get an overview of how well each descriptor performed, we averaged the ACC of the 11 classifiers-based models trained on each descriptor and the results were shown in Figure 2A. All of these descriptors achieved reasonable performance on the A2OM, C2OM and G2OM datasets, with average ACC in the ranges of 0.768–0.844, 0.747–0.825 and 0.755–0.826, respectively. However, the performance on the U2OM dataset was significantly lower, with average ACC in the range of 0.674–0.711. After evaluating the performance of 616 models on the independent testing datasets (Figure 2B), we observed that the average performance of each descriptor on the independent testing datasets was similar to that on the training datasets, with the U2OM dataset being the most challenging dataset. Specifically, the average ACC of the descriptors on the independent testing datasets ranged from 0.752 to 0.885 for A2OM, 0.736 to 0.877 for C2OM, 0.751 to 0.863 for G2OM and 0.695 to 0.791 for U2OM. Out of the 14 descriptors, DPCP_2, Kmer and PS2 performed the best on the training datasets, with ACC of 0.837–0.844 for A2OM, 0.822–0.825 for C2OM and 0.815–0.826 for G2OM. Notably, DPCP_2 and PS2 maintained a similar level of performance on the corresponding testing datasets. Since each of the descriptors captures different aspects of sequence information, we included all 14 conventional descriptors in the construction of an HDL framework.

### Evaluation of H2Opred for 2OM site prediction on the training and independent testing datasets

**Figure 3:**
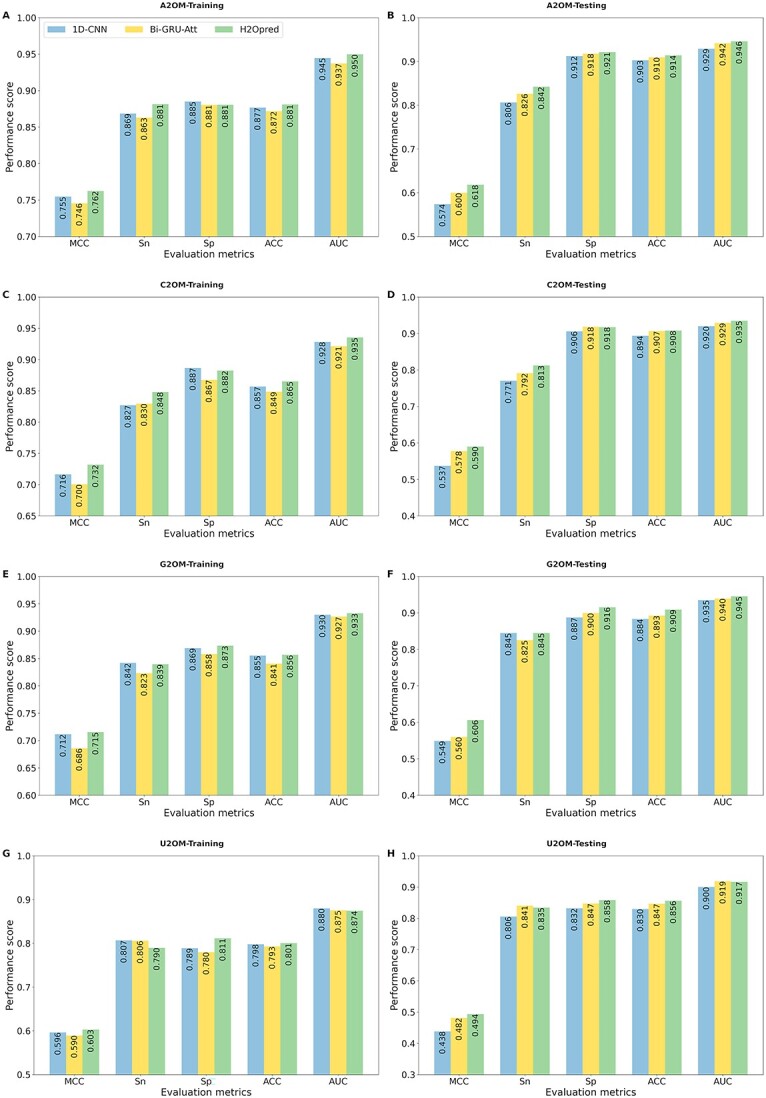
Comparison of nucleotide-specific H2Opred models and individual stacking models (1D-CNN and Bi-GRU-Att) on the training and testing datasets. Performance on the A2OM training (A) and testing (B) datasets, C2OM training (C) and testing (D) datasets, G2OM training (E) and testing (F) datasets, and U2OM training (G) and testing (H) datasets.

H2Opred was constructed using an HDL approach (see Materials and methods section). This approach combined stacked 1D-CNN blocks to learn effective feature representations from 14 conventional descriptors, and stacked Bi-GRU-Att blocks to learn feature representations from five NLP-based embeddings. These feature representations were then combined to make the final prediction. Figure 3 compares the performance of H2Opred with two individual approaches, 1D-CNN and Bi-GRU-Att. Among the 1D-CNN and Bi-GRU-Att models, 1D-CNN achieved consistently better performance on four training datasets, with improvement in MCCs of 0.09%, 1.60%, 2.60% and 0.06% for A2OM, C2OM, G2OM and U2OM, respectively. When combining these two models, H2Opred achieved MCC and ACC of 0.762 and 0.881 in A2OM, 0.732 and 0.865 in C2OM, 0.715 and 0.856 in G2OM and 0.603 and 0.801 in U2OM. Specifically, H2Opred outperformed 1D-CNN on three training datasets (A2OM, C2OM and G2OM), and achieved similar performance on the U2OM dataset. Overfitting occurs when the ML model learns the training data too well, and is unable to generalize to new data. To avoid overfitting, it is important to evaluate the model’s performance on the independent testing datasets.

We comprehensively evaluated the three deep learning models on four different testing datasets. In contrast to training performance, Bi-GRU-Att consistently outperformed 1D-CNN across all testing datasets, with improvement in MCCs of 2.57%, 4.04%, 1.09% and 4.31% for A2OM, C2OM, G2OM and U2OM, respectively, as shown in Figure 3. However, H2Opred significantly outperformed both 1D-CNN and Bi-GRU-Att on all four testing datasets. Specifically, H2Opred improved MCC by 1.84–4.41% in A2OM, 1.23–5.27% in C2OM, 4.64–5.72% in G2OM and 1.23–5.53% in U2OM compared with Bi-GRU-Att and 1D-CNN, respectively. Overall, H2Opred demonstrated superior performance compared with both 1D-CNN and Bi-GRU-Att, exhibiting consistent performance across both training and testing datasets. To highlight the advantages of H2Opred, we compared its performance against the top five conventional ML-based single-feature models for each of the four nucleotide-specific models. As illustrated in Figure S5, H2Opred achieved significantly better results than the conventional ML-based single-feature models on both training and independent datasets. These findings suggest that H2Opred possesses greater stability and generalizability across diverse data scenarios, because it can learn effective feature representations by combining conventional descriptors with NLP-based embeddings.

### Cross nucleotide-specific model validation

**Figure 4:**
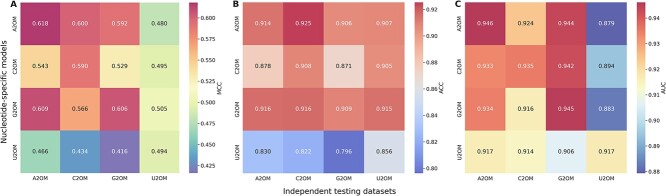
Heat map illustrating the cross-nucleotide model validation based on the testing datasets. Each nucleotide-specific model is evaluated with its own dataset as well as the remaining three nucleotide-specific datasets. The performance is assessed using three metrics: (A) Mathews correlation coefficient (MCC), (B) accuracy (ACC) and (C) area under the receiver operating characteristic curve (AUC).

We assessed the transferability of nucleotide-specific models to different testing datasets. We examined whether A2OM model is transferable to other types (C2OM, G2OM and U2OM), and vice versa, as shown in Figure 4. When the A2OM testing dataset was evaluated with the other three trained models (G2OM, C2OM and U2OM), they achieved the ACC in the range of 0.830 to 0.916, the AUC in the range of 0.917 to 0.934 and the MCC in the range of 0.466 to 0.609. Similarly, the trained A2OM, G2OM and U2OM models achieved the ACC in the range of 0.822 to 0.925, the AUC in the range of 0.914 to 0.924 and the MCC in the range 0.434 to 0.600 on the C2OM testing dataset; the trained A2OM, C2OM and U2OM models achieved the ACC in the range of 0.796 to 0.906, the AUC in the range of 0.906 to 0.944 and the MCC in the range of 0.416 to 0.592 on the G2OM testing dataset; and the trained A2OM, C2OM and G2OM models achieved the ACC in the range of 0.905 to 0.915, the AUC in the range of 0.879 to 0.894 and the MCC in the range of 0.480 to 0.505 on the U2OM testing dataset. Overall, these analyses highlight that a nucleotide-specific model can be effectively transferrable to other nucleotides with satisfactory accuracy. Previous studies [10, 25] have highlighted those four different nucleotides shares similar motif patterns at 2OM sites. This prompted us to investigate whether developing a generic model by integrating four different nucleotides could enhance the prediction performance compared with individual nucleotide-specific models.

### Development of a generic model

**Figure 5:**
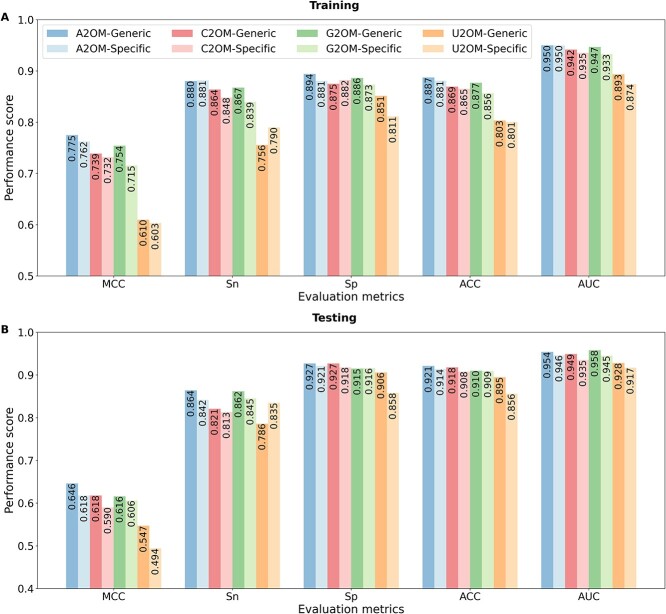
Performance of the generic model for each of the four nucleotides is compared with the corresponding nucleotide-specific models. The comparison is shown for both the (A) training dataset and (B) testing dataset.

To develop a generic model, we integrated the four nucleotide-specific datasets and eliminated redundant sequences using CD-HIT, resulting in 4256 positive and 4255 negative samples. The generic model was then built using the same approach outlined for the nucleotide-specific models. The results demonstrated that the generic model achieved MCC, Sn, Sp, ACC and AUC of 0.719, 0.840, 0.876, 0.858 and 0.937, respectively, during training. The corresponding values on the testing dataset were 0.602, 0.828, 0.918, 0.910 and 0.946, respectively (Figure S6). We compared the generic model’s performance with the individual nucleotide-specific models in two ways: (i) we averaged the performance of four nucleotide-specific models and compared it with the generic model. Figure S6 shows that the generic approach consistently outperformed the nucleotide-specific approach in terms of MCC, ACC and AUC on both training and testing datasets. (ii) We categorized generic model’s predictions based on nucleotide specificity and compared them with the individual nucleotide-specific models. Figure 5 illustrates that the generic model marginally improved MCC, ACC and AUC on G2OM and U2OM, and significantly improved on the other two nucleotide training datasets. However, the generic model significantly outperformed all four nucleotide-specific models on the independent testing datasets. In addition, we developed 154 conventional models, as previously mentioned for the development of nucleotide-specific models, to investigate whether any of these models surpassed the performance of the HDL-based generic model. As illustrated in Figure S7, the HDL-based generic model consistently outperformed the top five best models based on the MCC among the conventional models on both training and independent datasets, highlighting the significance of the HDL approach implemented in H2Opred.

Unlike individual nucleotides, 2OM occurs on the ribose moiety rather than specific modification sites like 5-methyl cytosine (m5C) and 6-methyl adenosine (m6A). As 2OM is not directly linked to nucleotide bases, using a generic dataset instead of nucleotide-specific datasets would be more appropriate since such datasets lack biological significance. Overall, the enhanced performance of our generic model can be attributed primarily to the larger training dataset.

### Comparison of H2Opred models with the state-of-the art predictors on the independent testing datasets

To further demonstrate the superiority of H2Opred models, we tried to compare them with four previously published methods: NmSEER v2.0, NmRF, DeepOMe and i2OM. However, we encountered challenges in extracting prediction results from the given sequence when using NmSEER v2.0 and DeepOMe. Therefore, we excluded these two methods from our comparative analysis and focused on NmRF and i2OM. Upon scrutinizing the performance of these two state-of-the-art predictors, we found that NmRF performed poorly, with results approaching random prediction levels (Figure S8). This makes NmRF unsuitable for genome-wide predictions. The primary reason for this subpar performance is that NmRF was trained on a substantially different dataset with a smaller number of samples than our models and i2OM. Comparing nucleotide-specific H2Opred models and i2OM directly is more straightforward and insightful because they were trained on the same datasets, allowing for a more meaningful evaluation of their respective performances. However, the generic H2Opred model was trained on different and larger training dataset. Owing to the challenges in accessing results from the i2OM web server, we utilized the standalone program (https://github.com/yangmoo1010/i2OM) to compute the results.

**Figure 6:**
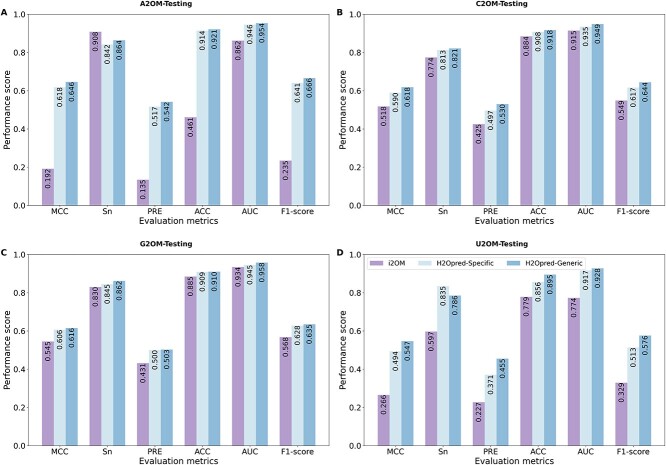
Comparison of the performance of different H2Opred models and the state-of-the-art i2OM predictor on four testing datasets. (A) A2OM, (B) C2OM, (C) G2OM and (D) U2OM.

Figure 6 clearly shows that the generic H2Opred model achieved the best performance across all testing datasets. Compared with i2OM, the generic H2Opred model demonstrated significant improvements across several key metrics, including MCC, ACC, AUC and F1-score. Specifically, on the A2OM testing dataset, the generic H2Opred model achieved 45.44% improvement in MCC, 46.00% in ACC, 9.16% in AUC and 43.16% in F1-score. Similarly, on the C2OM testing dataset, the generic H2Opred model outperformed i2OM with improvements of 10.05% in MCC, 3.31% in ACC, 3.40% in AUC and 9.52% in F1-score. The advantages of the generic H2Opred model were evident on the G2OM testing dataset, where it achieved improvements of 7.03% in MCC, 2.49% in ACC, 2.35% in AUC and 6.74% in F1-score. On the U2OM testing dataset, the generic H2Opred model achieved with remarkable improvements of 28.10% in MCC, 11.64% in ACC, 15.42% in AUC and 24.73% in F1-score. Furthermore, the generic H2Opred model consistently outperformed the nucleotide-specific H2Opred models across all datasets, demonstrating the significant of a larger training on performance improvement. These results highlight the stability and excellence of proposed H2Opred models, making H2Opred as an effective and valuable computational tool for the precise identification of RNA 2OM sites.

### Visualization of learned features

**Figure 7:**
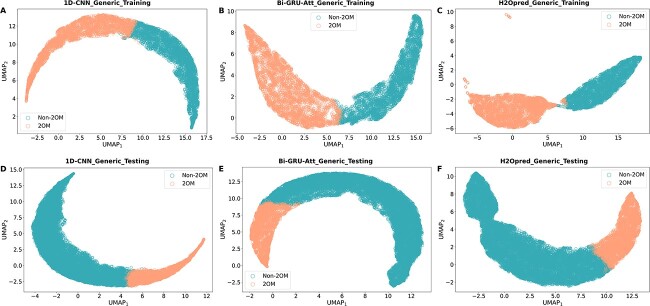
UMAP visualization of the learned feature representations of three models (1D-CNN, Bi-GRU-Att and H2Opred) on the generic training (A–C) and independent testing (D–F) datasets.

In this section, we focused on the best generic model and visualized the learned features extracted by 1D-CNN, Bi-GRU-Att and H2Opred to understand the abstractions generated by these deep learning architectures. We employed UMAP, a novel dimensionality reduction technique, to reduce the original features into a 2D vector. Figure 7 shows that the feature representations learned by Bi-GRU-Att showed minimal overlap between 2OM and non-2OM sites, while those learned by 1D-CNN showed more overlapping samples. However, the feature representations learned by H2Opred showed a clear separation of 2OM and non-2OM samples on the generic training dataset. This suggests that H2Opred is capable of extracting the most informative and discriminative features through 1D-CNN and Bi-GRU-Att. The feature representation patterns observed in the training dataset follows a similar on the testing dataset for three deep learning architectures, demonstrating that the model is able to learn robust representations of the 2OM and non-2OM sites from training datasets, which can be generalized to new data.

Through UMAP visualization, we demonstrated that conventional feature descriptors and NLP-based embeddings can be mapped into meaningful representations through the HDL approach. This suggests that H2Opred can capture complex relationships between different features, which is not possible with conventional ML-based single-feature models. Consequently, H2Opred consistently outperformed other deep learning-based models and conventional ML-based single-feature models, making it a promising tool for identifying 2OM sites in the human genome.

### Web server implementation

H2Opred, a user-friendly web server for predicting RNA 2OM sites, is now freely available at https://balalab-skku.org/H2Opred/. The website also provides access to all training and testing datasets employed in this study. To use H2Opred, users can either upload a file containing multiple FASTA sequences or input one or more query sequences in FASTA format. The input sequence must be in the FASTA format and exactly 41 bp long. Users are then be prompted to select their model of choice (N2OM, A2OM, G2OM, C2OM or U2OM) before job submission. If no model is selected, the N2OM model will be employed automatically. Upon successful job completion, the results are presented on a dedicated interface, allowing users to easily view and analyze the findings. Additionally, users have the option to download the results in CSV format for future reference.

## CONCLUSION

2OM is a common post-transcriptional modification with important implications for various biological functions. Understanding its distribution within RNA can provide insights into its mechanism of action. In this study, we introduced a novel HDL model called H2Opred, to accurately identify 2OM sites from primary RNA sequences. We first identified 14 conventional descriptors and evaluated their discriminative patterns using the 11 ML-based classifiers. We then used stacked 1D-CNN to learn the feature representations from these conventional descriptors. Next, we used NLP-based embeddings derived from RNA sequence and trained using stacked Bi-GRU-Att to capture more diverse facets of feature representations. Finally, we integrated these two distinct feature representations to make final prediction. Rigorous cross-validation and independent testing on different datasets showed that H2Opred achieved a more balanced performance and consistently outperformed the two deep learning architectures as well as several hundreds of ML-based single-feature models.

In contrast to individual nucleotides, which have specific modification sites like m5C and m6A, 2OM occurs on the ribose moiety, a structural component of RNA. Therefore, developing 2OM site prediction model using the nucleotide-specific datasets lacks biological significance. Indeed, our cross-nucleotide model analysis demonstrated the transferability of nucleotide-specific models across other nucleotide datasets, suggesting that a generic model can be developed by integrating all nucleotide-specific datasets. To this end, we developed a generic model that showed significant improvement on both training and testing datasets compared with the nucleotide-specific models. In the H2Opred webserver, we have incorporated both the four nucleotide-specific models and a generic model. Notably, each of these models demonstrated superior performance over the existing predictor i2OM across all evaluation metrics on the independent testing datasets. This suggests that our HDL framework integrated conventional descriptors with NLP-based embeddings to significantly improve prediction performance. To make H2Opred widely accessible and user-friendly, we have launched a dedicated web server at https://balalab-skku.org/H2Opred/. We believe that this online platform will be invaluable for researchers seeking to accurately predict 2OM sites in human RNA, advancing scientific progress in this field. The current approach can be extended to other RNA post-transcriptional prediction sites [47], RNA subcellular localization [48] and peptide therapeutic function prediction [27].

H2Opred has shown significant promise in predicting 2OM sites, but there are areas for further refinement. The proposed model was trained on a larger dataset than existing methods. However, we can further enhance the model performance by incorporating new data as it becomes available. We integrated conventional descriptors with NLP-based embeddings through an HDL framework, but future work could explore other methodologies, such as contrastive learning [49, 50], ensemble or stacking approaches based on conventional ML classifiers [51], hybrid feature approach [52], feature representation learning [53] and iterative feature refinement [54, 55]. Beyond conventional descriptors and NLP-based embeddings, the topological structure of RNA sequences also plays an important role in defining their functions. To improve feature representation, we could incorporate predicted structural information within the HDL framework. Currently, H2Opred is still a ‘black box’, limiting its ability to explain its decisions. In the future, we aspire to develop a model that is not only accurate but also interpretable, providing tangible and actionable biological insights.

**Key Points**

- We introduced a novel HDL model called H2Opred for accurately identifying 2’-O-methylation (2OM) sites in human RNA.
- H2Opred used a combination of stacked 1D-CNN and Bi-GRU-Att blocks to learn effective feature representation from 14 conventional descriptors and five NLP-based embeddings, respectively. Subsequently, these representations were combined to make the final prediction.
- A novel generic model has been developed, driven by cross-nucleotide model analysis, significantly outperforming nucleotide-specific models and the existing predictor when tested on large samples of an independent dataset.
- H2Opred is now available as a web server at https://balalab-skku.org/H2Opred/, providing an invaluable tool for predicting 2OM sites in human RNA.

## Supplementary Material

H2Opred_SI_bbad476

## Data Availability

Web server and all the processed data used in this study can be accessed via https://balalab-skku.org/H2Opred/.

## Author Biographies

**Nhat Truong Pham** is a Ph.D. student at the Computational Biology and Bioinformatics Laboratory, Department of Integrative Biotechnology, Sungkyunkwan University, Republic of Korea. His research interests include artificial intelligence, bioinformatics, computational biology and medicine, deep learning, machine learning, signal processing and XAI and optimization.

**Rajan Rakkiyappan** is a professor in the Department of Mathematics at Bharathiar University in Tamil Nadu, India. His research interests include neural networks, stability theory, data-driven machine learning, bioinformatics and computational biology.

**Jongsun Park** is a co-CEO and CTO of Infoboss Inc, Republic of Korea. His research interests include bioinformatics, genomics, deep learning, machine learning, bio big data, plant/insect taxonomy and phylogenetics.

**Adeel Malik** is a Research Professor at the Institute of Intelligence Informatics Technology, Sangmyung University, Seoul, Republic of Korea. His research interests include machine learning, computational biology and comparative genomics.

**Balachandran Manavalan** is an Assistant Professor at the Department of Integrative Biotechnology, Sungkyunkwan University, Republic of Korea. He is also an associate member of Korea Institute for Advanced Study, Republic of Korea. His research interests include artificial intelligence, bioinformatics, machine learning, big data and functional genomics.
