# Supplementary material for: H2Opred: a robust and efficient hybrid deep learning model for predicting 2’-O-methylation sites in human RNA
Source: Brief Bioinform. 2024 Jan 4;25(1):bbad476. doi: 10.1093/bib/bbad476 (PMC10768780; doi:10.1093/bib/bbad476)
Supplement: H2Opred_SI_bbad476 [file h2opred_si_bbad476.pdf]

Conventional descriptors

ENAC: ENAC represents an improved iteration of nucleic acid (NA) composition (NAC), achieved by employing a fixed-length window that consistently moves along the input RNA sequence from the N-terminus to the C-terminus. This approach is used to determine the occurrence of each nucleic acid type within the window. Specifically, the frequency (f) within a sliding window (SW) can be calculated as follows:

$$f(NA_i) = \frac{\sum(NA_i)}{SW}, \tag{1}$$

where NA_i represents nucleic acid types, namely adenine (A), cytosine (C), guanine (G), and uracil (U), denoted as $NA_i \in \{A, C, G, U\}$. The total count of a specific nucleic acid type, denoted as $\Sigma(NA_i)$, signifies the overall occurrences of that particular nucleic acid. It's crucial to note that the sum of frequencies for nucleic acids within an SW must equate to 1. For example, if the sliding window ($SW = 5$) comprises an RNA subsequence like [A, C, C, U, U], the resulting frequencies should be [0.2, 0.4, 0, 0.4], representing the occurrence percentages of nucleic acid types A, C, G, and U within the given sliding window, respectively.

PS2: PS2 denotes pairs of adjacent nucleotides in a pairwise fashion, encompassing combinations such as AA, GC, CG, GU, CA,…UU, totaling 16 distinct pairs [1]. These 16 pairs are then encoded into 16 binary bits, each falling within the set $\{0, 1\}$. For example, AG is represented as (0010000000000000), while GC is denoted as (0000000001000000). Consequently, an RNA sequence like AGGU is encoded as (001000000000000000000000001000000000000000010000).

CKSNAP: CKSNAP integrates the concepts of NAC and PS2 within the framework of nucleic acid pairs. Specifically, CKSNAP computes the frequencies of 16 nucleic acid pairs, where each pair is separated by a *k*-spaced nucleic acid, denoted as $k \in \{0, 1, 2, 3, 4, 5\}$. These 16 pairs align with those in PS2, encompassing combinations like AA, CC, GG, UA, CA, …, UU. Notably, when $k = 0$, the 16 pairs in CKSNAP are generated similarly to those in PS2. However, unlike PS2, CKSNAP calculates these 16

pairs based on NAC instead of encoding them into binary bits. In practical terms, for an RNA sequence of length L, the feature vector for each k-spaced scenario can be defined as follows:

$$CKSNAP = [f(NA_{xy})^{(i)}, f(NA_{xy})^{(i+1)}, ..., f(NA_{xy})^{(P)}], \quad (2)$$

where $f(NA_{xy})$ represents the frequency of the paired nucleic acid xy, and it can be computed as follows:

$$f(NA_{xy}) = \frac{\sum(NA_{xy})}{L-k+1}, \quad (3)$$

$NA_{xy} \in \{AA, AC, AG, AU, CA, \ldots, UU\}$, where $i = 1, 2, 3, \ldots, P$ with $P = 16$ represents the number of pairs, and $\Sigma(NA_{xy})$ denotes the total count of the paired nucleic acid xy in the given sequence.

PseEIIP: The method of using electron-ion interaction pseudo potential (EIIP) values was utilized to encode DNA or protein sequences. This approach involves evaluating the energy of delocalized electrons within nucleotide or amino acid sequences. When applied to RNA sequences, specific EIIP values are assigned: A is encoded with a value of 0.1260, C with 0.1340, G with 0.0806, and U (equivalent to T) with a value of 0.1335, as reported in [2].

Using the EIIP encoding as a foundation, the PseEIIP encoding vector is derived by integrating the principles of EIIP and ENAC encodings in the following manner:

$$PseEIIP = [(EIIP_{xyz} \times f(NA_{xyz}))^{(i)}, ..., (EIIP_{xyz} \times f(NA_{xyz}))^{(T)}]. \quad (4)$$

In equation (4), $EIIP_{xyz} = EIIP_x + EIIP_y + EIIP_z$, $f(NA_{xyz})$ represents the normalized frequency of the trinucleotide, where $NA_{xyz} \in \{AAA, AAC, AAG, AAU, \ldots, UUU\}$. Here, $i = 1, 2, 3, \ldots, T$ with T being the total number of trinucleotides. It is essential to emphasize that $f(NA_{xyz})$ is computed using NAC for the respective trinucleotide.

Zcurve: Zcurve encompasses data regarding the frequencies of phase-specific tri-nucleotides, as elucidated in a prior study [3]. The computation of Zcurve information is carried out as follows:

$$\begin{cases} i_{RS}^{z} = (p^{z}(RSA) + p^{z}(RSG)) - (p^{z}(RSC) + p^{z}(RSU)) \\ i_{RS}^{z} = (p^{z}(RSA) + p^{z}(RSC)) - (p^{z}(RSU) + p^{z}(RSG)), \\ i_{RS}^{z} = (p^{z}(RSA) + p^{z}(RSU)) - (p^{z}(RSC) + p^{z}(RSG)) \end{cases} \quad (5)$$

where $R\ or\ S \in \{A, U, G, C\}; z = 1, 2, 3$.

Kmer: The Kmer encoding algorithm is grounded in nucleic acid composition within the context of *k* neighboring nucleic acids. In practical terms, Kmer computation involves varying values of $k \in \{1, 2, 3, 4, 5\}$, representing mononucleotide, dinucleotide, trinucleotide, tetranucleotide, and pentanucleotide, respectively. . This approach yields a 1364-dimensional (1364D) feature vector (resulting from $4^1 + 4^2 + 4^3 + 4^4 + 4^5$). For an RNA sequence of length L, the frequencies of $k = 2$ can be calculated as follows:

$$f(NA_{km}) = \frac{\sum(NA_{km})}{L}, \quad (6)$$

where $NA_{km} \in \{AA, AC, AG, AU, CA, \ldots, UU\}$. Here, $\Sigma(NA_{km})$ represents the count of different types of *k*-neighboring nucleic acids within the provided sequence.

RCKmer: RCKmer represents a distinct form of Kmer generated by eliminating the reverse complement k-mers for each variety of *k*-neighboring nucleic acids in the provided sequence [4, 5]. For example, when $k = 2$, specific 2-mers such as 'CU', 'GG', 'GU, 'UC', 'UG', and 'UU' are excluded, leaving behind 10 discriminatively unique 2-mers in the RCKmer set.

DPCP_1: There exist 22 distinct physicochemical properties associated with dinucleotides in RNA sequences [1, 6]. Most of these properties, including attributes like shift, chemical enthalpy, slide, physical enthalpy, stacking energy, rise, chemical entropy, physical entropy, chemical hydrophilicity, physical hydrophilicity, tilt, roll, chemical free energy, physical free energy, and twist, can be extracted for any dinucleotide sequence. However, some properties are unique to specific dinucleotides, such as keto (GU), adenine content, purine (AG) content, guanine content, GC content, cytosine content,

and thymine content. It is important to note that the default value for these properties is zero for all dinucleotides, except for the specific ones mentioned.

The DPCP_1 can be defined as:

$$DPCP\_1 = [(PCP_{xy}^{(i)} \times f(xy))^{(j)}, \ldots, (PCP_{xy}^{(i+1)} \times f(xy))^{(j+1)}, \ldots, (PCP_{xy}^{(N_{PCP})} \times f(xy))^{(N_D)}], \quad (7)$$

where x and y are nucleotide symbols representing adenine (A), cytosine (C), guanine (G), or uracil (U). The variable i ranges from 1 to N_{PCP}, indicating the total number of physicochemical properties. Similarly, j ranges from 1 to N_D, representing the number of dinucleotides. $f(xy)$ signifies the normalized frequency of the dinucleotide xy, and $PCP^{(i)}$ denotes the *i*-th physicochemical property. Consequently, the output of DPCP_1 results in a 352D vector (16×22), representing the various dinucleotides and physicochemical properties, respectively.

DPCP_2: DPCP_2 is an upgraded form of DPCP_1. When considering an RNA sequence with a length of L, the DPCP_2 can be described as:

$$DPCP\_2 = [PCP^{(i)}(NA_x^{(m)} NA_y^{(n)}), \ldots, PCP^{(i+1)}(NA_x^{(m+1)} NA_y^{(n+1)}), \ldots, PCP^{(N_{PCP})}(NA_x^{(L-1)} NA_y^{(L)})], \quad (8)$$

where $NA_x, NA_y \in \{AA, AC, AG, AU, CA, \ldots, UU\}$, $m = 1, 2, \ldots, (L-1)$, $n = (m+1)$, $(m+2), \ldots, L$, and $PCP^{(i)}(NA_x^{(m)} NA_y^{(n)})$ is the *i*-th physicochemical property of the dinucleotide, $NA_x^{(m)} NA_y^{(n)}$.

NCP: Among the four fundamental nucleotide types (A, C, G, and U), there exist three categories of chemical attributes: ring structure (comprising purines A, G, and pyrimidines C, U), hydrogen bond (strong in C, G, and weak in A, U), and functional group (including amino A, C, and keto G, U). Each property has two classifications, signifying specific nucleotide types distinguished by their unique chemical properties.

Utilizing chemical properties, each nucleic acid is represented by a set of three coordinates (X, Y, Z) as follows:

$$X_{NA_i} = \begin{cases} 0 & if \quad NA_i \in \{U, C\} \\ 1 & if \quad NA_i \in \{G, A\} \end{cases};$$

$$Y_{NA_i} = \begin{cases} 0 & if \quad NA_i \in \{U, G\} \\ 1 & if \quad NA_i \in \{C, A\} \end{cases}; \tag{9}$$

$$Z_{NA_i} = \begin{cases} 0 & if \quad NA_i \in \{G, C\} \\ 1 & if \quad NA_i \in \{U, A\} \end{cases}.$$

As a result, A, C, G, and U can be encoded by (1, 1, 1), (0, 1, 0), (1, 0, 0), and (0, 0, 1), respectively.

BPF: BPF, commonly referred to as one-hot encoding, is a popular technique utilized for encoding various biological sequences such as DNA, RNA, peptides, and proteins. Specifically in the context of RNA sequences, each nucleotide is represented by a 4D binary vector: A (1000), C (0100), G (0010), and U (0001). Consequently, for an RNA sequence of length L, the resulting vector is flattened, possessing dimensions of $4 \times L$.

ASLPN: The ASLPN method combines two techniques: adaptive skipped dinucleotide composition (ASDC) and local position-specific dinucleotide frequency (LPSDF). Here is a brief overview of each:

ASDC encoding is an advanced version of dinucleotide composition, integrating the *k*-skip-*n*-gram model. Specifically, it utilizes *k*-skipped nucleotides during *n*-gram model computation, incorporating both distance and composition information. Due to the exponential increase in feature vector dimensions with higher $n - gram$ values, this method focuses on $n = 2$ (dinucleotide) analysis. For an RNA sequence of length L, the output feature vector of ASDC is represented as follows:

$$ASDC = [f_1(NA_{xy}), f_2(NA_{xy}), ..., f_i(NA_{xy}), ..., f_{16}(NA_{xy})]. \tag{10}$$

Here, $i = 1, 2, \ldots, 16$, and $NA_{xy} \in \{AA, AU, AC, AG, \ldots, UU\}$. The frequency of occurrence for all potential dinucleotides, considering k-skipped nucleotides, is calculated as follows:

$$f_i(NA_{xy}) = \frac{\sum_{k=1}^{L-1} NA_{xy}^{(k)}}{\sum_{i=1}^{16}\sum_{k=1}^{L-1} NA_{xy}^{(k)}}. \quad (11)$$

LPSDF, a method of dinucleotide composition, determines the frequency of dinucleotides formed by the nucleotide at a specific position and the preceding position within an RNA sequence. The occurrence frequencies of this dinucleotide at the *i*-th position can be calculated as follows:

$$fi = \frac{\sum (NA_x^{(i-1)} NA_y^{(i)})}{SW}. \quad (12)$$

Here, SW represents the length of the subsequence $\{NA_x^{(1)}, NA_x^{(2)}, \ldots, NA_x^{(i)}\}$, where $SW = L - i$. In equation (12), L denotes the length of the provided RNA sequence, and NA_x, NA_y belong to the set $\{A, C, G, U\}$.

MMNF: The MMNF is formed through the integration of multivariate mutual information (MMI) and accumulated nucleotide frequency (ANF). The procedure for deriving MMI and ANF is outlined as follows:

To calculate the MMI encoding [7], we employ the frequencies of *k*-mers where $k \in \{2, 3\}$. Subsequently, the mutual information corresponding to these frequencies can be computed as follows:

For $k = 2$,

$$MI_2(NA_{xy}) = f(NA_{xy}) \ln\left(\frac{f(NA_{xy})}{f(NA_x) f(NA_y)}\right). \quad (13)$$

And for $k = 3$,

$$MI_3(NA_{xyz}) = f(NA_{xy}) \ln\left(\frac{f(NA_{xy})}{f(NA_x) f(NA_y)}\right) + \frac{f(NA_{xz})}{f(NA_z)} \ln\left(\frac{f(NA_{xz})}{f(NA_z)}\right) - \frac{f(NA_{xyz})}{f(NA_{yz})} \ln\left(\frac{f(NA_{xyz})}{f(NA_{yz})}\right).$$

(14)

Here, NA_x, NA_y, NA_z belong to the set $\{A, C, G, U\}$, and $f(NA_x), f(NA_y), f(NA_z)$ represent their respective frequencies in the RNA sequence. $f(NA_{xy}), f(NA_{xz})$ indicate the frequencies of '2-mer' like NA_{xy} (e.g., AC, CG, GU, UA, etc.), and $f(NA_{xyz})$ represents the frequency of '3-mer' such as NA_{xyz} (e.g., AAC, CCG, GGU, UUA, etc.).

The ANF results from blending the cumulative NAC with NCP. The cumulative frequency is outlined as follows:

$$\rho = \frac{1}{N_{NA_x}} \sum_{i}^{L} f(NA_x), \tag{15}$$

where L represents the length of the RNA sequence, N_{NA_x} denotes the occurrence count of nucleotides NA_x ($NA_x \in \{A, C, G, U\}$) in the prefix sequence $[1, 2, \ldots, i]$. The $f(NA_x)$ values are encoded by using binary bits in the set $\{0, 1\}$. For example, considering the RNA sequence 'ACGUUGCA', the resulting NCP encoding would be {(1, 1, 1), (0, 1, 0), (1, 0, 0), (0, 0, 1), (0, 0, 1), (1, 0, 0), (1, 1, 1), (0, 1, 0)}. Simultaneously, the accumulated frequency (ρ) values of 'A' are 1 and 0.25 at positions 1 and 8, 'C' has values 0.50 and 0.29 at positions 2 and 7, 'G' exhibits 0.33 at positions 3 and 6, and 'U' shows 0.25 and 0.40 at positions 4 and 5, respectively. Incorporating these values, the final ANF encoding becomes {(1, 1, 1, 1), (0, 1, 0, 0.50), (1, 0, 0, 0.33), (0, 0, 1, 0.25), (0, 0, 1, 0.40), (1, 0, 0, 0.33), (0, 1, 0, 0.29), (1, 1, 1, 0.25)}. This integration enhances the NCP encoding by incorporating crucial long-range sequential order information.

DBE: The DBE descriptor captures positional information regarding each dinucleotide in the sequence. There are 16 possible dinucleotides, and each is represented by a 4D 0/1 vector within the descriptor. For instance, AA is represented as (0,0,0,0), AU as (0,0,1,1), CC as (0,1,0,1), and so on, with UU represented as (1,1,1,1). Consequently, the DBE produces a 160D (40×4) 0/1 vector for the given sequence.

NLP-based embeddings

We extracted several NLP-based embeddings from RNA sequences to enhance the richness of feature representation for predicting 2'-O-methylation (2OM) sites. These embeddings, including DNABERT, Seq2Vec, Word2Vec, FastText, and GloVe, capture both embedding information and preserving the inherent sequence order characteristics of RNA. Here is an overview of the different features of each NLP-based embedding.

DNABERT: DNABERT [8] is a pre-trained model tailored for processing DNA sequences. It uses a bidirectional encoder representation from the Transformer model, which allows it to learn relationships between nucleotides in both directions of sequence. DNABERT employs a Kmer-based tokenizer to tokenize DNA sequences into discrete units. Subsequently, these tokens undergo processing through 12 Transformer blocks to derive comprehensive representations. While DNABERT was primarily developed for DNA sequences, its applicability to RNA sequences has been demonstrated in [8, 9]. Specifically, RNA sequences differ from DNA sequences by just one base (thymine to uracil), while the syntax and semantics largely remain unchanged. As a result, DNABERT can be used to process 2OM RNA sequences by simply replacing the nucleotide "U" with the nucleotide "T" before tokenization. Notably, when provided with an RNA sequence, DNABERT can generate a feature matrix that not only contains embeddings (768D) but also valuable temporal-sequential ordering information.

Seq2Vec: Seq2Vec, a model designed for converting sequence-to-vector, takes inspiration from ELMo (embedding from language model). The ELMo framework encompasses two primary components: a pair of bidirectional long short-term memory (Bi-LSTM) layers and a character-aware CNN layer. The integration of the character-aware CNN layer serves to distill insights from individual characters, thereby shaping the token representations. These token representations undergo further refinement through two successive Bi-LSTM layers, facilitating the acquisition of context-independent embeddings. Notably, within the ELMo architecture, all layers are harmonized via a linear layer positioned on top, resulting in the ultimate representations. For a given RNA

sequence, it orchestrates the generation of a feature matrix endowed with embeddings (1024D) containing crucial temporal-sequential ordering information.

Word2Vec: Word2Vec [10] is a language model (LM) conceptualized by Google, which combines the strengths of the continuous bag-of-words (CBOW) and continuous skip-gram models. This fusion enhances the precision of word representations. Through exposure to extensive text corpora, Word2Vec demonstrates its ability to ingeniously map words onto dense vector representations that intricately encode semantic relationships throughout the lexicon. Here, we harnessed the potential of Word2Vec to distill enriching embedding features from RNA sequences, which resulted in a feature matrix encompassing 512D embeddings.

FastText: FastText [11] is a text classification model developed by the Facebook Research team in 2016. It is known for its simplicity and effectiveness, and is often used as a foundational model for various text classification tasks. FastText operates by incorporating n-gram features and using a hierarchical SoftMax loss to construct a linear classifier. This unique approach allows FastText to capture statistical associations between words and their contextual information, leading to its exceptional performance in text classification. Here, we leveraged FastText to generate numerical feature vectors from RNA sequences. These embeddings are numerical representations of words or phrases that are carefully crafted to capture their semantic meaning. By employing FastText embedder, we obtained a feature matrix consisting of 512D embeddings along with sequence ordering information for the RNA sequences.

GloVe: GloVe [12] is a word representation model that employs global word-word co-occurrence counts to capture statistical information about the relationships between words. Unlike models that rely on local context windows, GloVe harnesses the full corpus to learn significant substructures within the word vector domain. Here, GloVe was used to extract valuable features from RNA sequences, which resulted in feature matrix consisting of 512D that captures both the sematic and sequential information.

Supplementary Table

Table S1. Parameter search range employed in developing H2Opred framework.

**Parameter**	**Searching range**
Conv1D layer 1	{64, 128, 256, 512}
Conv1D layer 2	{32, 64, 128}
Conv1D layer 3	{16, 32, 64}
FC layer 1	{32, 64, 128, 256, 512}
Bi-GRU layer 1	{64, 128, 256}
Bi-GRU layer 2	{32, 64, 128}
FC layer 2	{32, 64, 128, 256, 512}
FC layer 3	{32, 64, 128, 256, 512}
Learning rate	{5e-4, 1e-4, 5e-3, 1e-3, 1e-2}
Dropout rate	{0.1, 0.2, 0.3, 0.4, 0.5}
Batch size	{16, 32, 64, 128, 256}

Supplementary Figures

Figure S1. Performance comparison among 154 conventional models based on Matthews correlation coefficient (MCC), accuracy (ACC), and area under the receiver operating characteristic curve (AUC) on the A2OM training and testing datasets.

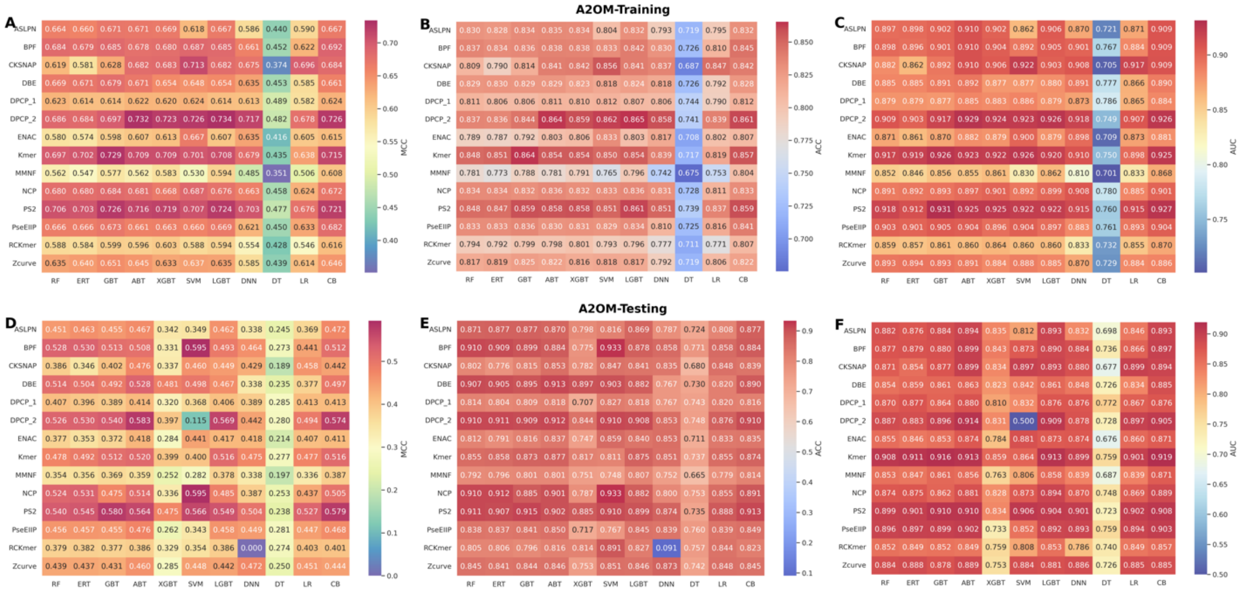

Figure S2. Performance comparison among 154 conventional models based on the MCC, ACC, and AUC on the C2OM training and testing datasets.

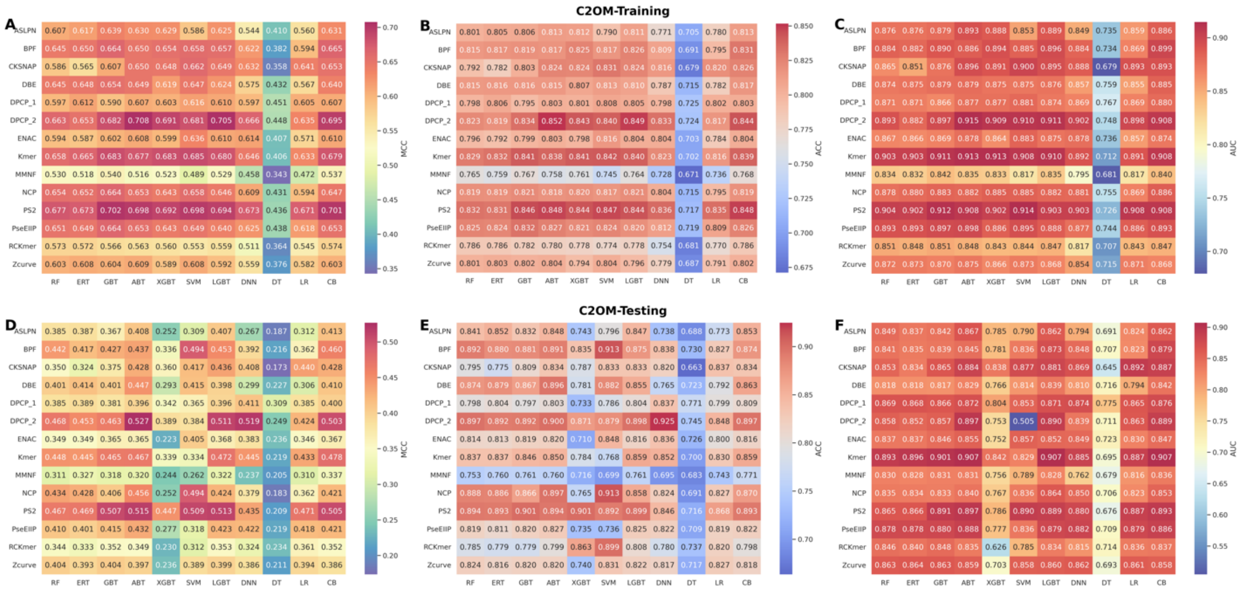

Figure S3. Performance comparison among 154 conventional models based on the MCC, ACC, and AUC on the G2OM training and testing datasets.

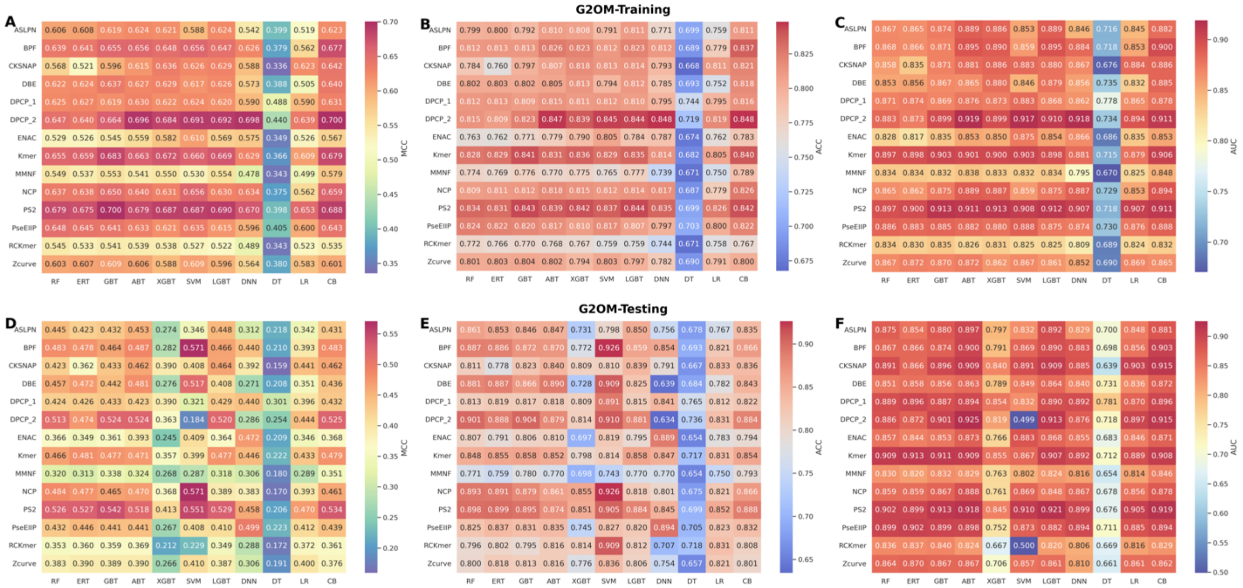

Figure S4. Performance comparison among 154 conventional models based on the MCC, ACC, and AUC on the U2OM training and testing datasets.

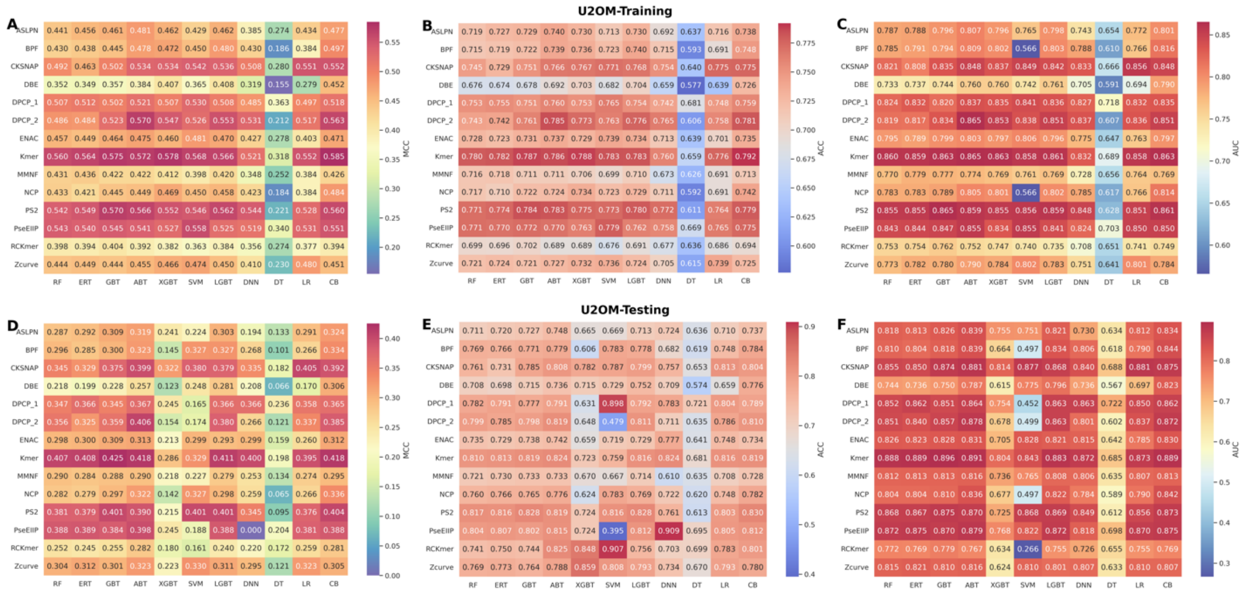

Figure S5. Performance comparison between H2Opred nucleotide-specific models and top five conventional classifiers on the training and testing datasets: (A-B) A2OM, (C-D) C2OM, (E-F) G2OM, and (G-H) U2OM.

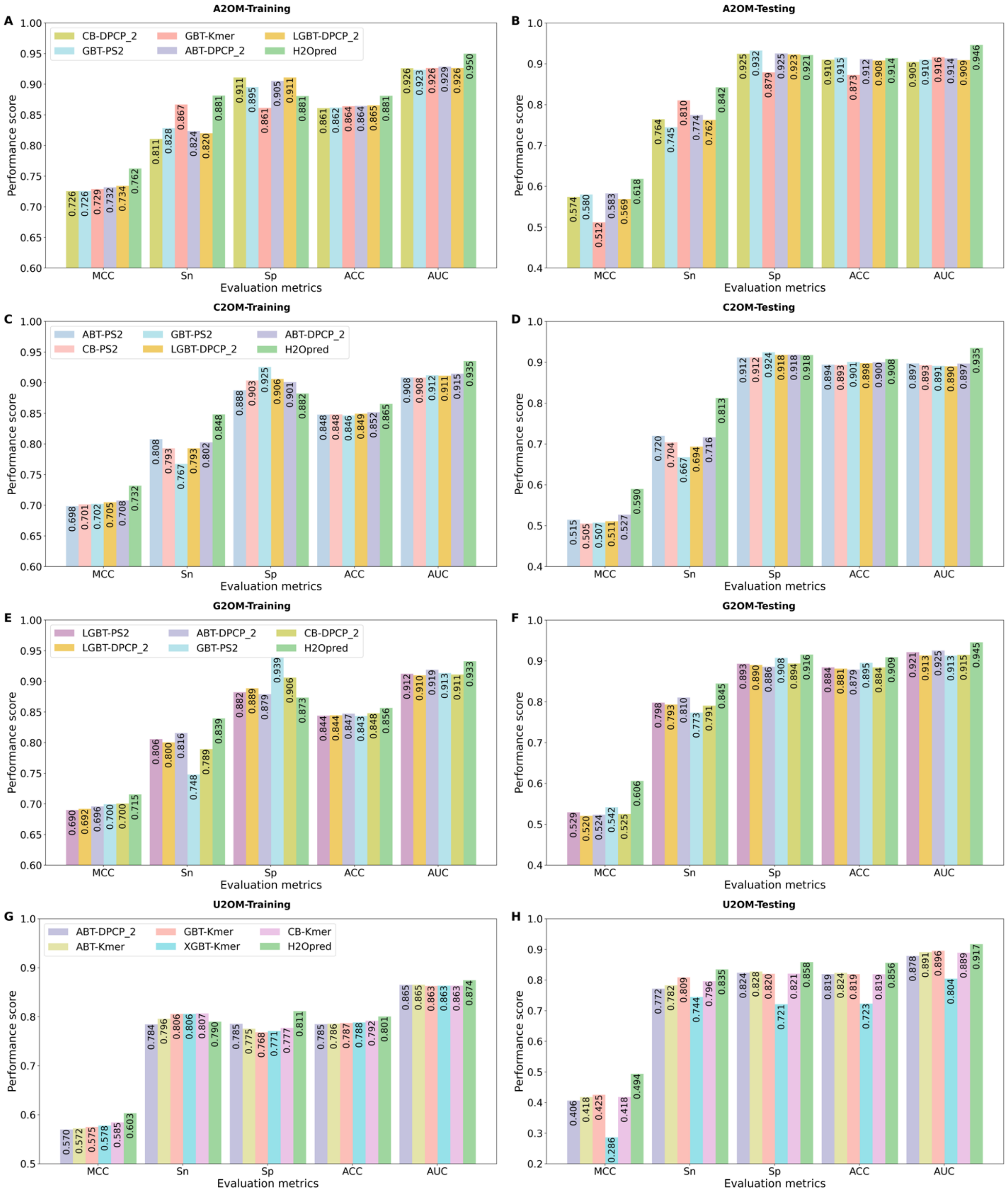

Figure S6. Comparison of performance between the average performance of the nucleotide-specific H2Opred models and the generic H2Opred model on the training and independent testing datasets.

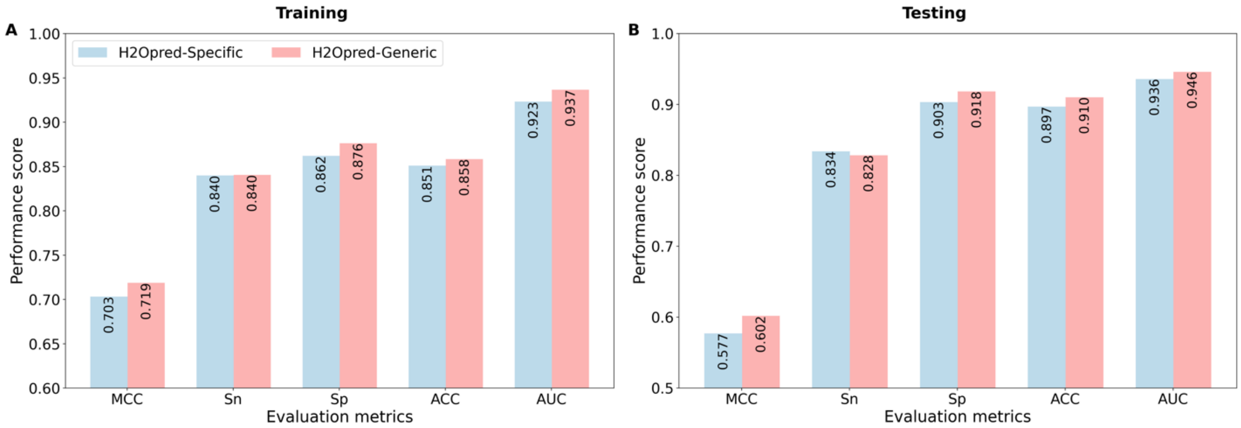

Figure S7. (A-B) Performance comparison among 154 conventional models based on the MCC on the generic training and testing datasets. **(C-D)** Performance comparison between the generic H2Opred model and the top five best conventional models on the generic training and testing datasets.

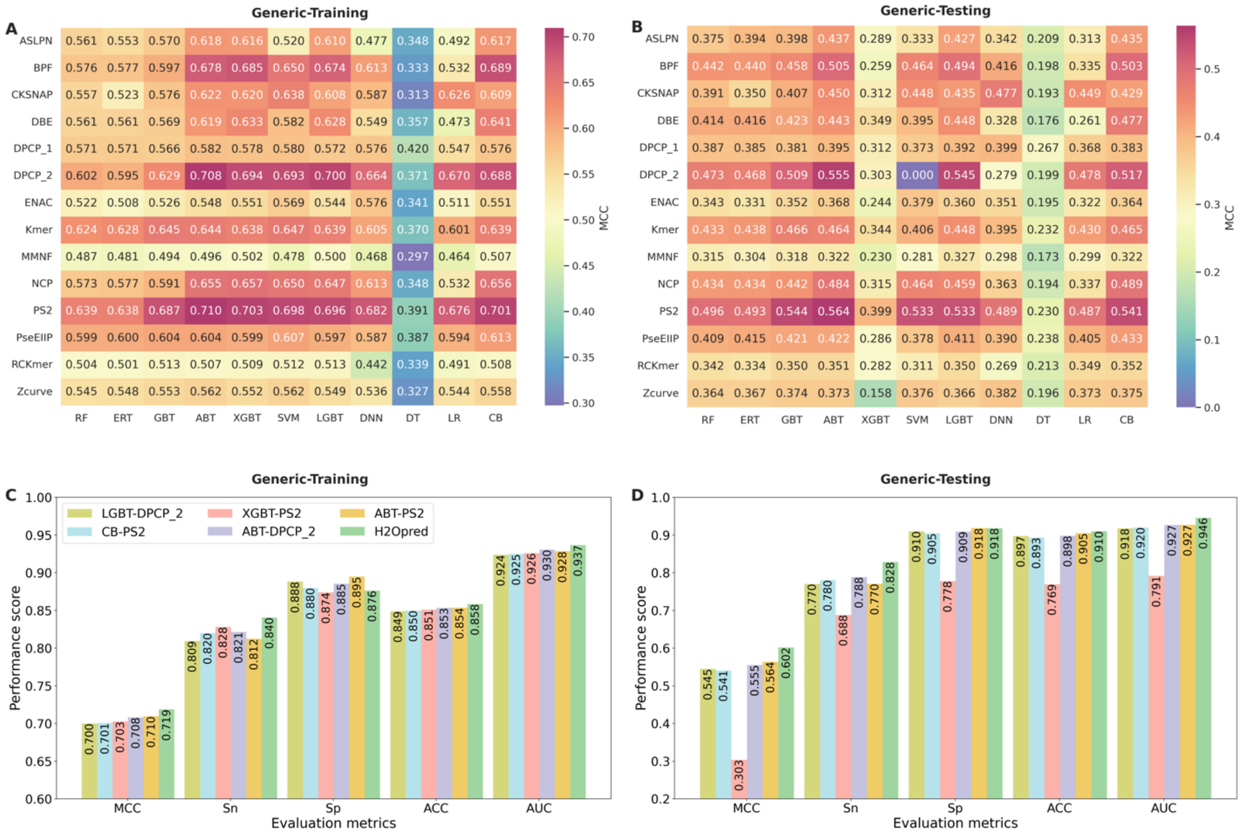

Figure S8. Performance comparison between H2Opred models and the state-of-the-art predictors on the independent testing datasets.

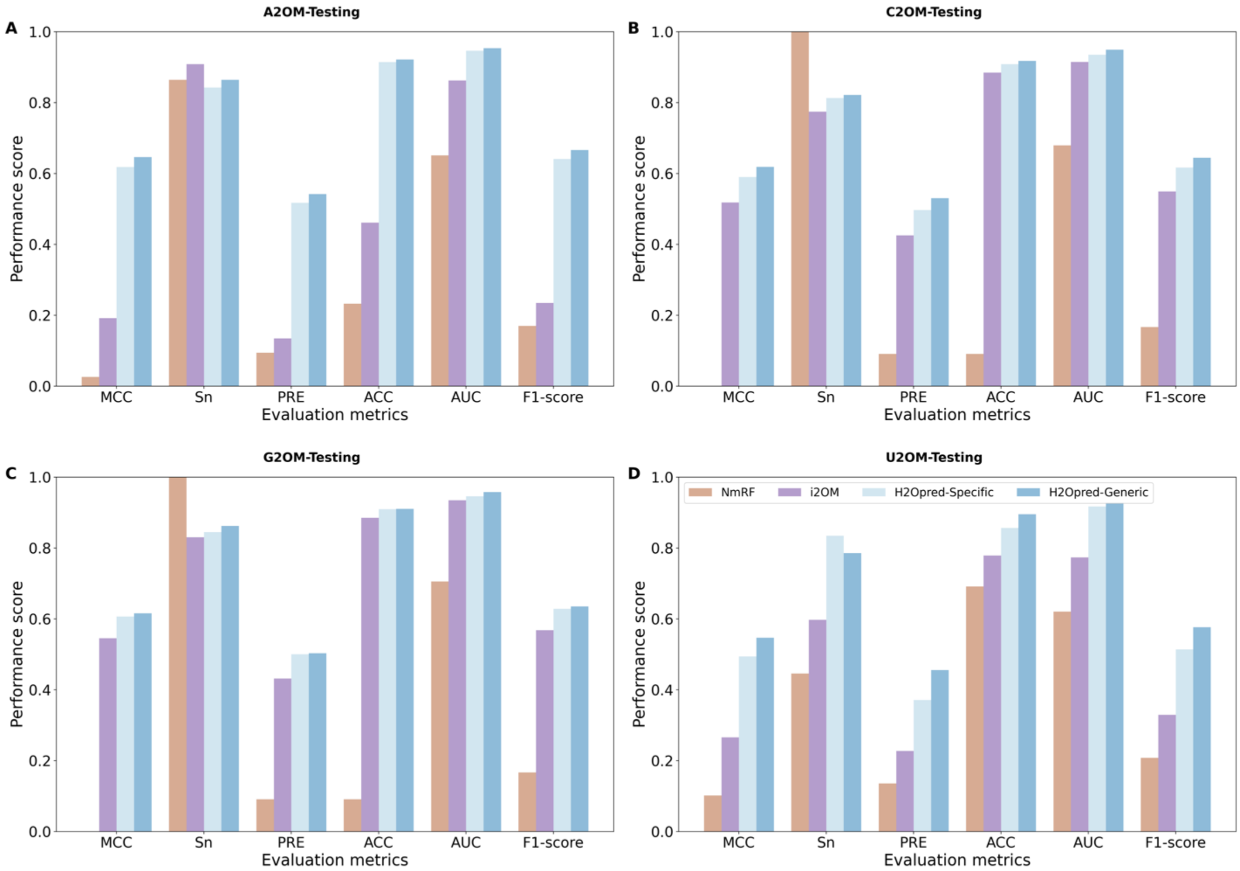

References

1. Liu B, Gao X, Zhang H. BioSeq-Analysis2.0: an updated platform for analyzing DNA, RNA and protein sequences at sequence level and residue level based on machine learning approaches, Nucleic Acids Res 2019;47:e127.
2. Nair AS, Sreenadhan SP. A coding measure scheme employing electron-ion interaction pseudopotential (EIIP), Bioinformation 2006;1:197-202.
3. Gao F, Zhang CT. Comparison of various algorithms for recognizing short coding sequences of human genes, Bioinformatics 2004;20:673-681.
4. Gupta S, Dennis J, Thurman RE et al. Predicting human nucleosome occupancy from primary sequence, PLoS Comput Biol 2008;4:e1000134.
5. Noble WS, Kuehn S, Thurman R et al. Predicting the in vivo signature of human gene regulatory sequences, Bioinformatics 2005;21 Suppl 1:i338-343.
6. Chen Z, Liu X, Zhao P et al. iFeatureOmega: an integrative platform for engineering, visualization and analysis of features from molecular sequences, structural and ligand data sets, Nucleic Acids Res 2022;50:W434-W447.
7. Wei L, Su R, Luan S et al. Iterative feature representations improve N4-methylcytosine site prediction, Bioinformatics 2019;35:4930-4937.
8. Ji Y, Zhou Z, Liu H et al. DNABERT: pre-trained Bidirectional Encoder Representations from Transformers model for DNA-language in genome, Bioinformatics 2021;37:2112-2120.
9. Yamada K, Hamada M. Prediction of RNA-protein interactions using a nucleotide language model, Bioinform Adv 2022;2:vbac023.
10. Mikolov T, Chen K, Corrado G et al. Efficient estimation of word representations in vector space, arXiv preprint arXiv:1301.3781 2013.
11. Joulin A, Grave E, Bojanowski P et al. Bag of tricks for efficient text classification, arXiv preprint arXiv:1607.01759 2016.
12. Pennington J, Socher R, Manning CD. Glove: Global vectors for word representation. In: Proceedings of the 2014 conference on empirical methods in natural language processing (EMNLP). 2014, p. 1532-1543.
